# Field performance on grain yield and quality and genetic diversity of overwintering cultivated rice (*Oryza sativa* L.) in southwest China

**DOI:** 10.1038/s41598-021-81291-8

**Published:** 2021-01-19

**Authors:** Yongshu Liang, Wenbin Nan, Xiaojian Qin, Hanma Zhang

**Affiliations:** Chongqing Key Laboratory of Molecular Biology of Plant Environmental Adaptations, Chongqing Normal University, Chongqing, 401331 China

**Keywords:** Plant breeding, Plant reproduction, Plant sciences

## Abstract

**U**nderstanding the field performance on grain yield and quality and the genetic diversity of overwintering (OW) cultivated rice (*Oryza sativa* L.) across main crop (MC) and ratooning crop (RC) is the premise to make strategies for the future OW rice variety improvement in rice production. The present field experiments were conducted in RC of 2016, in MC of both 2017 and 2018, and RC in 2019 to identify genotypes OW rice that perform stable in terms of grain yield and quality across different climate conditions. The grain yield plant^-1^ (GYP) and its components in six genotypes of OW rice exhibited significant difference across the 4 years (P ≤ 0.05), the maximum GYP in OW6 rice was harvested (60.28 g) in MC of 2017, but the minimum GYP in OW1 rice was harvested (33.01 g) in MC of 2018. Within six genotypes of OW rice, four grain shape traits displayed a relative small significant difference, four grain quality traits exhibited a relative small significant difference except for chalkiness rate (CR), there 226 pairs of significant *PCC* values between GYP and its components were calculated in all tested rice and varied from six in OW6 to eleven in OW1, there 130 pairs of significant *PCC* values among the four grain shape traits were calculated and ranged from twenty-one in OW1, 3, 5 to twenty-three in OW2, there 118 pairs of significant *PCC* values among the four grain quality traits were calculated and ranged from seventeen in OW2 to twenty-three in OW1. The numbers, directions, and size of *PCC* values for the grain yield and quality characters in all tested rice displayed a series of irregular variations. Six genotypes of OW rice were apparently distinguished by employing 196 pairs of simple-sequence repeats (SSRs) markers and exhibited abundant genetic diversity at the DNA level. Data from this study provide an extensive archive for the future exploration and innovation of overwintering cultivated rice variety.

## Introduction

Rice (*Oryza sativa* L.) is the predominant staple food throughout the world, feeding more than half of the world’s population ^1^. Particularly in China, rice has been being played an important role in ensuring national food security ^2^. Currently, China’s rice production has been facing many challenges including the decreasing cultivated paddy, rapid population growth, and the increasing shortage of labor ^3–5^. More seriously, the recent increases in labor wage have significantly increased the cost of food production and lowered agricultural competitiveness in the global market, which indirectly raises food security concerns in China^6,7^. Being faced with the decreasing rice production, overwintering (perennial) cultivated rice could be planted once a year and harvested many times in rice production and exhibited a ray of hope in alleviating these conflicting demand for Chinese people’s rice consumption and being considered as a very economical strategy involved in sustaining rice grain-yield through labor cost saved ^8,9^. On the other hand, growing overwintering rice also can prevent soil desertification and protect soil resources in the ecosystem restoration ^10^. Consequently, it is urgent to develop overwintering cultivated rice for the food security in China. Up to now, the study on genetics and breeding of overwintering rice has been performed since Tao & Sripichitt (2000) firstly reported the successful hybridization cross between *Oryza sativa* L. and *Oryza longistaminata* through the technology of embryo rescuing^11^. Liang et al^12^ located three major quantitative trait loci (QTLs) (qOW2, qOW3 and qOW6) underlying overwintering traits using Chinese perennial Dongxiang wild rice. Hu et al^13^ and Zhang et al^14^ have succeeded in developing perennial rice through selecting O. *longistaminate* as gene donors. The field performance on both GYP and its components in perennial rice genotypes were examined and easily influenced by both genotype and external environment factors^15,16^. Up to now, the genes or QTLs underlying perennial trait from O. *longistaminata* have been successfully transferred into common cultivated rice and even perennial rice variety would be commercially released to farmers as result of reduced labor costs ^17^. Meanwhile, the genes (QTLs) underlying overwintering traits was also hidden in the existing cultivated rice variety ^18^. However, the field performance on both grain yield and quality and even genetic diversity of overwintering cultivated rice has been largely unknown. Currently, it is necessary to perform the study on field performance variations in grain yield and quality of overwintering cultivated rice across different environmental conditions before we integrate multi-genes (QTLs) underlying overwintering traits into a novel overwintering rice variety adapted to Chongqing in southwest China.

To this end, six genotypes of overwintering (OW) rice identified during the natural snowy winter season in Chongqing, southwest China, were grown to evaluate the field performance variations in grain yield and quality under different climate condition. The present objectives were (i) to evaluate field performance variations in grain yield and quality of OW rice across main crop (MC) and ratooning crop (RC), (ii) to identify genotypes OW rice that perform stable in terms of grain yield and quality across different climate conditions, and examine the genetic diversity of all tested rice using SSR marker, and (iii) to identify excellent OW cultivated rice resources involved in elucidating the molecular mechanisms about OW characteristics and the development of novel OW cultivated rice variety.

## Materials and methods

### Study site description

Four cycle field experiments across RC in 2016, MC in 2017, MC in 2018 and RC in 2019 were conducted in a randomized complete block design with three replicates at the Biotechnology Testing Station of Chongqing Normal University (CQNU), Chongqing (29°32′ N, 106°32′ E), southwest China. Six genotypes of overwintering (OW) cultivated rice (*Oryza sativa* L.) could germinate through natural snowy winter, flowering and being harvested (Supplementary Fig. 1 a-d), designated as OW1, OW2, OW3, OW4, OW5, and OW6 below, respectively, and were descended from six hybrid combinations of ‘Wu913/Zhong12121', ‘Wu913/Zhong12135', ‘Wuyunjing24/Zhong12135',‘Wumingjing24/Wx1337', ‘Wumingjing/L1', and ‘Wu913/910’.

In RC of 2016, rice stub of all tested rice were retained with 30 cm, survived through natural cold-winter season, germinated and flowering and being harvested for the grain yield and quality evaluated. In MC of 2017, seeds collected from six OW cultivated rice were sown on 15 March 2016 and 35-day-old seedlings of all tested rice were transplanted into four-row plots with six plants per row, 20 cm between plants within each row, and 23 cm between rows. Similarly, a parallel test was performed in both MC of 2018 and RC of 2019, on 10 March 2018, seeds of all tested rice were sown and 35-day-old seedlings of all OW cultivated rice were also transplanted. In RC of 2019, all OW cultivated rice could germinate after being harvested for grain yield and quality evaluated (Supplementary Fig. 2–4).

A special rice compound fertilizer (450 kg urea ha^-1^) occupied more than 45% of total nutrients was made up of 12% N, 18% P_2_O_5_, and 15% K_2_O and applied at the basal stage. Nitrogen (180 kg urea ha^-1^, 46% N) was applied 2 weeks after transplanting seedlings. The water management strategy adopted was shallow water at the transplanting stage and flooding midseason with drainage-reflooding-moist intermittent irrigation. Weed control, pest management, and disease treatment were carried out to avoid grain yield loss.

### DNA extraction and PCR amplification

Six genotypes of OW cultivated rice and two sequenced rice varieties of Nipponbare and 93-11 were sampled at stage of rice tillers. The rice genomic DNA was extracted referred to the method cetyltrimethylammonium bromide (CTAB) described by Nei ^19^. A total of 196 pairs of simple-sequence repeats (SSR) markers were employed to evaluate the genetic diversity of six genotypes of OW cultivated rice and two sequenced rice (data not shown). PCR amplifications were performed referred to the protocol described by Greer et al. ^20^. DNA products were separated by 8% polyacrylamide gel electrophoresis (PAGE).

### Measurements

#### Grain yield

A random sample of five plants per plot within a single genotype of OW rice for each replication across RC of 2016, MR of both 2017 and 2018, and RC of 2019 was collected to measure the following phenotypic values according to the method described by Shen ^21^: HD, days to heading; PH, plant height (cm); PP, panicles plant^-1^; PL, panicle length (cm); FGP, fulling grains panicle^-1^; EGP, empty grains panicle^-1^; SP, spikelets panicle^-1^; GW, 1000-grain weight (g), GYMP, grain yield of major panicle (g); and GYP, grain yield plant^-1^ (g) were measured. Grain shape traits including GL, grain length (mm); GW, grain width (mm); and GT grain thickness (mm) were measured with a Mitutoyo absolute digimatic caliper (Model 500-173). Three derived traits were calculated including GSR, grain setting rate (%); GSD, grain-setting density; and LWR, length to width ratio. The phenotypic data for each trait in five plants within individual genotype with three replicates were calculated for statistical analysis.

### Grain quality

Four grain quality traits including CR, chalkiness rate (%); AC, amylose content (%); GC, gel consistency (mm); and ADV, alkali digestion value (class) were measured at the Rice Product Quality Inspection & Supervision Testing Center, Ministry of Agriculture. China National Rice Research Institute (CNRRI), Hangzhou, China. Chalkiness version 2.0 software was used to measure CR according to the method described in NY14788 and GB/T17891-1999^22^; the spectrophotometric method according to the Chinese Ministry of Agriculture’s NY/T 2639-2014 was used to measure AC. Two methods described in GB/T22294-2008 and NY/T83-2017 were used to measure GC and ADV, respectively ^23^.

### Statistical analysis

All phenotypic data on grain yield and quality collected from all tested rice across RC in 2016, MC in 2017, MC in 2018, and RC in 2019 were juxtaposed in Microsoft Excel 2010 to perform the analysis on multiple comparison, Means ± SD and phenotypic correlation coefficient (PCCs) using the software DPS7.5 version and Graph PadPrism 5.0 version (GraphPad Software, San Diego, California, USA). The multiple comparison analysis was performed based on the Duncan’s new multiple range method at the 0.05 probability. Based on the PCR amplification of nine pairs of SSR primers with good polymorphism, 0 and 1 represented the non-amplified and amplified band were used to arrange the molecular data in Microsoft Excel 2010. The genetic similarity coefficient was calculated using the software NTSYSpc 2.1 version (Applied Biostatistics, Port Jefferson, New Yokr, USA).

## Results

### Climatic condition

**Figure 1:**
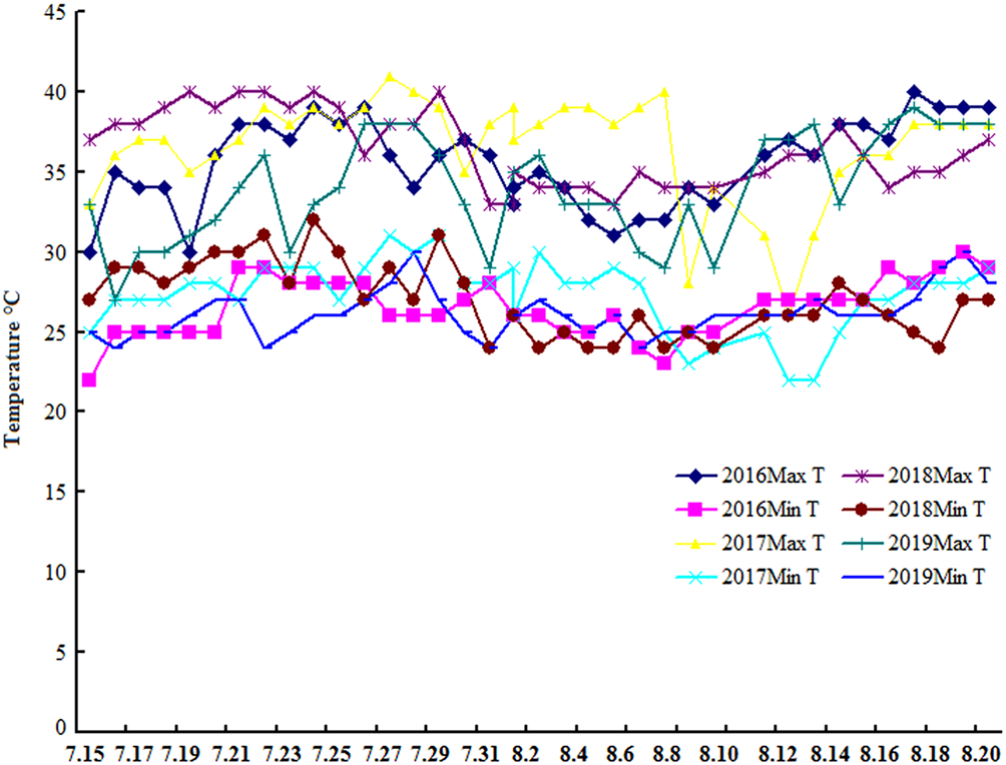
Temperature conditions during each ripering period for OW rice across the 4 years.

There was relatively small difference on average value both daily minimum temperature (Min T, °C) and daily maximum temperature (Max T, °C) during each ripening period from July 15 to August 20 across RC in 2016, MC in 2017, MC in 2018, and RC in 2019 (Table 1). The daily Min T (°C) almost exhibited nonsignificant difference across the 4 years (Fig. 1). However, the highest daily Max T (°C) during ripening period displayed a significant difference across the 4 years. For example, the highest daily Max T (°C) in MC of 2017 reached 40 °C on 7 August 2017. However, on 7 August 2019, the highest daily Max T (°C) in RC of 2019 was only 29 °C. There was significant difference on contemporaneous Max T, °C across the 4 years.

**Table 1:** Temperature conditions during each ripening period for OW rice across the 4 years.

Dates	RC in 2016 (°C)		MC in 2017 (°C)		MC in 2018 (°C)		RC in 2019 (°C)	
	Max T	Min T	Max T	Min T	Max T	Min T	Max T	Min T
7.15	30	22	33	25	37	27	33	25
7.16	35	25	36	27	38	29	27	24
7.17	34	25	37	27	38	29	30	25
7.18	34	25	37	27	39	28	30	25
7.19	30	25	35	28	40	29	31	26
7.20	36	25	36	28	39	30	32	27
7.21	38	29	37	27	40	30	34	27
7.22	38	29	39	29	40	31	36	24
7.23	37	28	38	29	39	28	30	25
7.24	39	28	39	29	40	32	33	26
7.25	38	28	38	27	39	30	34	26
7.26	39	28	39	29	36	27	38	27
7.27	36	26	41	31	38	29	38	28
7.28	34	26	40	30	38	27	38	30
7.29	36	26	39	31	40	31	36	27
7.30	37	27	35	28	37	28	33	25
7.31	36	28	38	28	33	24	29	24
8.1	33	26	39	29	33	26	35	26
8.2	35	26	38	30	34	24	36	27
8.3	34	25	39	28	34	25	33	26
8.4	32	25	39	28	34	24	33	25
8.5	31	26	38	29	33	24	33	26
8.6	32	24	39	28	35	26	30	24
8.7	32	23	40	25	34	24	29	25
8.8	34	25	28	23	34	25	33	25
8.9	33	25	34	24	34	24	29	26
8.10	34	26	37	26	35	26	35	26
8.11	36	27	31	25	35	26	37	26
8.12	37	27	26	22	36	26	37	26
8.13	36	27	31	22	36	26	38	27
8.14	38	27	35	25	38	28	33	26
8.15	38	27	36	27	36	27	36	26
8.16	37	29	36	27	34	26	38	26
8.17	40	28	38	28	35	25	39	27
8.18	39	29	38	28	35	24	38	29
8.19	39	30	38	28	36	27	38	30
8.20	39	29	38	29	37	27	38	28
Average	35.57	26.51	36.62	27.32	36.46	27.00	34.05	26.16

Daily maximum temperature (Max T, °C) and daily minimum temperature (Min T, °C) for each ripening period.

### GYP and its components in OW rice

**Figure 2:**
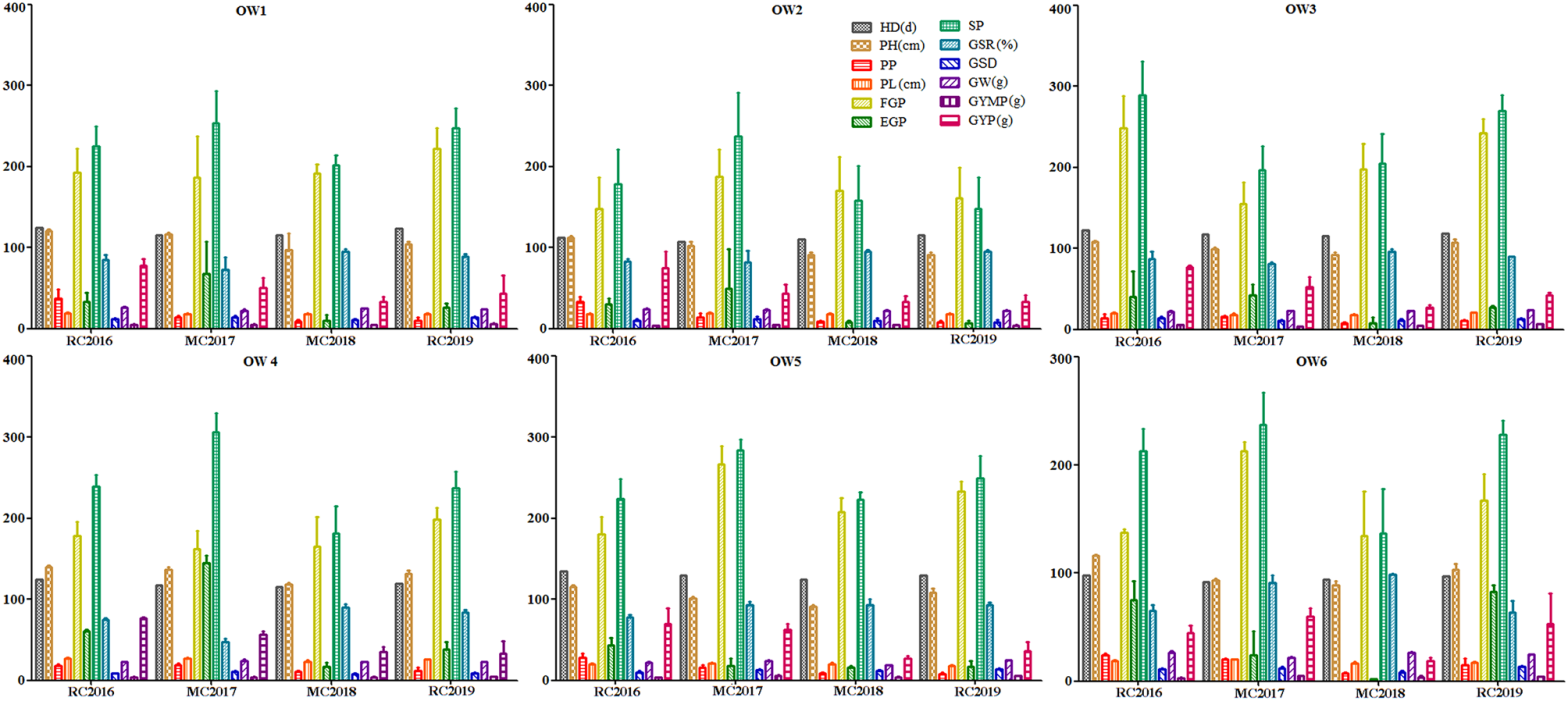
GYP and its components in six genotypes of OW rice.

The GYP and its eleven components in six genotypes of OW rice exhibited significant difference across RC in 2016, MC in 2017, MC in 2018, and RC in 2019 (Table 2). Within individual genotype of OW rice, the GYP almost displayed significant difference across the 4 years (Fig. 2). The maximum GYP in genotype of OW6 rice was harvested (60.28 g) in MC of 2017. However, the minimum GYP in genotype of OW1 rice was investigated (33.01 g) in MC of 2018. A series of significant differences on the eleven components of GYP were calculated in all tested rice across the 4 years. In particularly, the field performance values of HD, PH and PP in RC of both 2016 and 2019 were higher than those in MC of both 2017 and 2018 and displayed a relatively regular variation. However, the GSR in two genotypes of OW2 and OW3 were more than 80% across the 4 years. However, the GSR in the remaining four genotypes of OW rice partially displayed more than 80% across the 4 years. The remaining seven grain yield characters of PL, FGP, EGP, SP, GSD, GW and GYMP in all tested rice displayed a series of irregular significant difference across the 4 years. In summary, the field performances on GYP and its component in all tested rice expressed rather unstable under different climate conditions.

**Table 2:** GYP and its components in six genotypes of OW rice.

Genotypes	Crops	HD	PH	PP	PL	FGP	EGP	SP	GSR	GSD	GW	GYMP	GYP
		d	cm	n	cm	n			%		g		
OW1	RC2016	125^A^	121.00 ± 1.55^A^	37.25 ± 11.55^A^	18.79 ± 1.11 ^A^	192.25 ± 30.17 ^B^	32.75 ± 11.58 ^B^	225.00 ± 24.71 ^B^	85.03 ± 6.28 ^C^	11.93 ± 0.63 ^B^	25.76 ± 1.54 ^A^	4.46 ± 0.88 ^B^	77.50 ± 8.97 ^A^
OW1	MC2017	115 ^B^	116.60 ± 2.15 ^B^	13.60 ± 2.06 ^B^	17.82 ± 1.10 ^B^	186.20 ± 50.81 ^B^	67.60 ± 39.69 ^A^	253.80 ± 39.12 ^A^	73.26 ± 15.17 ^D^	14.21 ± 1.81 ^A^	22.14 ± 1.67 ^D^	4.30 ± 1.01 ^C^	50.76 ± 11.94 ^B^
OW1	MC2018	116 ^B^	97.40 ± 20.60 ^D^	9.20 ± 1.47 ^C^	18.19 ± 0.43 ^AB^	191.60 ± 11.22 ^B^	10.00 ± 6.57 ^D^	201.60 ± 12.91 ^C^	95.12 ± 3.05 ^A^	11.09 ± 0.72 ^B^	25.35 ± 0.14 ^B^	4.64 ± 0.24 ^B^	33.01 ± 6.60 ^D^
OW1	RC2019	124 ^B^	104.13 ± 3.43 ^C^	9.75 ± 4.26 ^C^	17.75 ± 1.06 ^AB^	221.75 ± 25.35 ^A^	26.00 ± 5.48 ^C^	247.75 ± 23.74 ^A^	89.38 ± 2.42 ^B^	13.93 ± 0.56 ^A^	24.24 ± 0.16 ^C^	5.58 ± 0.75 ^A^	43.54 ± 22.25 ^C^
OW2	RC2016	112 ^AB^	112.20 ± 2.54 ^A^	33.00 ± 6.51 ^A^	17.67 ± 1.73 ^B^	147.60 ± 39.44 ^D^	30.40 ± 6.67 ^B^	178.00 ± 42.92 ^B^	82.58 ± 3.66 ^B^	9.95 ± 1.42 ^B^	23.54 ± 1.14 ^A^	3.30 ± 0.90 ^C^	74.90 ± 20.59 ^A^
OW2	MC2017	107 ^B^	102.70 ± 4.29 ^B^	14.17 ± 4.39 ^B^	19.21 ± 0.66 ^A^	188.00 ± 33.13 ^A^	49.25 ± 49.02 ^A^	237.25 ± 53.77 ^A^	81.79 ± 14.85 ^D^	12.30 ± 2.53 ^A^	22.63 ± 1.46 ^B^	4.25 ± 0.75 ^A^	43.05 ± 11.33 ^B^
OW2	MC2018	110 ^AB^	91.40 ± 2.33 ^C^	8.40 ± 1.85 ^C^	17.97 ± 0.56 ^B^	170.80 ± 41.47 ^B^	7.80 ± 2.14 ^C^	158.60 ± 42.50 ^C^	95.50 ± 1.31 ^A^	10.00 ± 2.54 ^AB^	22.48 ± 0.16 ^AB^	4.31 ± 0.47 ^A^	33.33 ± 7.07 ^C^
OW2	RC2019	115 ^A^	91.00 ± 2.97 ^C^	8.00 ± 2.20 ^C^	18.15 ± 0.69 ^AB^	161.00 ± 38.03 ^C^	7.00 ± 2.58 ^C^	148.00 ± 38.97 ^C^	95.18 ± 1.64 ^A^	8.21 ± 2.37 ^C^	22.35 ± 0.20 ^AB^	3.99 ± 0.35 ^B^	32.99 ± 8.69 ^C^
OW3	RC2016	123 ^A^	108.00 ± 1.29 ^A^	13.97 ± 4.79 ^B^	20.07 ± 0.81 ^A^	248.72 ± 39.68 ^A^	40.03 ± 31.73 ^A^	288.75 ± 42.13 ^A^	86.59 ± 9.62 ^AB^	14.36 ± 1.94 ^A^	21.66 ± 1.04 ^B^	5.45 ± 0.71 ^B^	76.54 ± 2.79 ^A^
OW3	MC2017	118 ^A^	98.98 ± 2.27 ^B^	15.80 ± 1.21 ^A^	18.25 ± 1.64 ^A^	155.00 ± 26.62 ^C^	42.00 ± 13.78 ^A^	197.00 ± 29.19 ^C^	80.70 ± 2.61 ^B^	10.73 ± 0.82 ^C^	22.73 ± 0.58 ^AB^	3.53 ± 0.47 ^D^	52.59 ± 11.95 ^B^
OW3	MC2018	116 ^A^	92.10 ± 3.06 ^B^	7.40 ± 1.20 ^D^	18.45 ± 0.78 ^A^	197.40 ± 31.48 ^B^	8.00 ± 6.45 ^C^	205.40 ± 35.68 ^C^	96.38 ± 2.77 ^A^	11.11 ± 1.68 ^C^	22.56 ± 0.28 ^AB^	4.51 ± 0.38 ^C^	27.04 ± 2.78 ^D^
OW3	RC2019	119 ^A^	107.75 ± 3.25 ^A^	11.00 ± 1.00 ^C^	20.63 ± 0.13 ^A^	242.50 ± 17.50 ^A^	27.50 ± 1.50 ^B^	270.00 ± 19.00 ^B^	89.80 ± 0.16 ^AB^	13.09 ± 0.84 ^A^	23.60 ± 0.31 ^A^	6.89 ± 0.14 ^A^	42.24 ± 3.38 ^C^
OW4	RC2016	125 ^A^	140.00 ± 1.63 ^A^	17.50 ± 2.86 ^A^	27.55 ± 0.29 ^A^	179.00 ± 16.33 ^B^	60.50 ± 2.04 ^B^	239.5 ± 14.29 ^B^	74.53 ± 2.37 ^C^	8.69 ± 0.43 ^C^	22.80 ± 0.49 ^B^	4.17 ± 0.48 ^B^	76.53 ± 1.45 ^A^
OW4	MC2017	118 ^B^	137.10 ± 3.06 ^A^	18.80 ± 2.11 ^A^	27.23 ± 0.65 ^B^	161.80 ± 23.03 ^C^	144.80 ± 8.93 ^A^	306.60 ± 22.91 ^A^	47.52 ± 4.41 ^D^	11.26 ± 0.83 ^A^	24.35 ± 1.31 ^A^	4.15 ± 0.48 ^B^	56.75 ± 3.78 ^B^
OW4	MC2018	116 ^B^	118.70 ± 1.94 ^B^	10.40 ± 1.50 ^B^	23.52 ± 1.23 ^A^	165.00 ± 36.66 ^BC^	17.00 ± 5.25 ^D^	182.00 ± 33.52 ^C^	90.00 ± 4.43 ^A^	7.74 ± 1.43 ^D^	22.56 ± 0.07 ^B^	3.74 ± 0.77 ^C^	35.52 ± 6.26 ^C^
OW4	RC2019	120 ^A^	131.63 ± 4.22 ^A^	11.50 ± 4.15 ^B^	25.65 ± 0.71 ^A^	199.00 ± 14.04 ^A^	38.00 ± 9.19 ^C^	237.00 ± 21.25 ^B^	84.13 ± 2.78 ^B^	9.26 ± 1.06 ^B^	22.75 ± 0.11 ^B^	4.66 ± 0.42 ^A^	33.31 ± 15.48 ^C^
OW5	RC2016	135 ^A^	115.70 ± 1.46 ^A^	28.00 ± 5.20 ^A^	19.68 ± 0.82 ^B^	180.33 ± 21.79 ^D^	43.83 ± 8.41 ^A^	224.17 ± 24.32 ^C^	78.16 ± 2.57 ^B^	10.27 ± 1.32 ^D^	22.51 ± 0.39 ^B^	3.75 ± 0.28 ^B^	69.75 ± 19.53 ^A^
OW5	MC2017	130 ^BC^	101.70 ± 1.70 ^B^	16.00 ± 3.46 ^B^	21.49 ± 0.66 ^A^	266.57 ± 22.25 ^A^	17.60 ± 9.58 ^B^	284.17 ± 13.68 ^A^	93.62 ± 3.90 ^A^	13.23 ± 0.79 ^B^	23.84 ± 1.72 ^A^	5.73 ± 0.67 ^A^	62.29 ± 7.13 ^B^
OW5	MC2018	125 ^C^	91.08 ± 2.01 ^C^	8.40 ± 1.36 ^C^	19.77 ± 2.17 ^B^	207.60 ± 17.21 ^C^	15.94 ± 1.67 ^C^	222.80 ± 9.33 ^C^	93.22 ± 7.07 ^a^	11.40 ± 1.28 ^C^	25.95 ± 0.15 ^C^	3.84 ± 0.43 ^B^	27.30 ± 3.05 ^D^
OW5	RC2019	130 ^AB^	108.67 ± 4.70 ^AB^	8.00 ± 2.16 ^C^	17.99 ± 0.70 ^C^	233.00 ± 12.19 ^B^	17.00 ± 6.68 ^BC^	250.00 ± 27.53 ^B^	93.14 ± 2.86 ^A^	13.92 ± 0.64 ^A^	25.11 ± 0.03 ^A^	5.78 ± 0.28 ^A^	35.85 ± 11.66 ^C^
OW6	RC2016	98 ^A^	116.50 ± 0.41 ^A^	24.50 ± 1.22 ^A^	18.95 ± 0.45 ^B^	138.00 ± 2.45 ^C^	75.50 ± 17.55 ^B^	213.50 ± 20.00 ^B^	65.34 ± 4.98 ^C^	11.24 ± 0.79 ^C^	26.45 ± 1.59 ^A^	3.16 ± 0.27 ^C^	44.79 ± 6.60 ^B^
OW6	MC2017	92 ^B^	93.10 ± 2.21 ^C^	20.60 ± 0.73 ^B^	19.96 ± 0.54 ^A^	213.20 ± 8.25 ^A^	24.40 ± 22.16 ^C^	237.60 ± 30.01 ^A^	90.82 ± 7.32 ^B^	11.88 ± 1.27 ^B^	22.08 ± 0.79 ^B^	4.70 ± 0.37 ^A^	60.28 ± 7.06 ^A^
OW6	MC2018	94 ^AB^	89.00 ± 3.56 ^C^	7.00 ± 1.36 ^D^	16.75 ± 1.64 ^D^	135.00 ± 40.47 ^C^	2.00 ± 0.25 ^D^	137.00 ± 40.80 ^C^	98.54 ± 1.29 ^A^	8.18 ± 1.63 ^D^	26.38 ± 0.43 ^AB^	3.89 ± 0.94 ^B^	18.54 ± 3.05 ^C^
OW6	RC2019	97 ^A^	103.50 ± 5.12 ^B^	15.00 ± 5.89 ^C^	17.11 ± 0.83 ^C^	167.33 ± 24.09 ^B^	82.67 ± 6.60 ^A^	228.33 ± 13.02 ^A^	64.14 ± 10.14 ^C^	13.38 ± 1.04 ^A^	24.57 ± 0.14 ^AB^	3.97 ± 0.55 ^B^	52.73 ± 28.72 ^B^

Values followed by different letter are significantly different at 5% (capital) probability levels, respectively.

### *PCCs* between GYP and its components in OW rice

A total of 226 pairs of significant *PCC* values between GYP and its eleven components were calculated in six genotypes of OW rice across RC in 2016, MC in 2017, MC in 2018, RC in 2019 (Table 3). Among them, in RC of 2016, there 29 pairs of significantly positive *PCC* values were observed in all tested rice and ranged from one pair in OW3 to nine pairs in OW1. The GYP was significantly positive correlated with HD, PH, PP, PL, FGP, EGP, SP, GSD, and GYMP in genotype of OW1, the GYP was only positively correlated with PP in genotype of OW3. In MC of 2017, there 25 pairs of significantly positive *PCC* values in six genotypes of OW rice ranged from three pairs in OW6 and OW5 to seven pairs in OW1. GYP in genotype of OW1 was positively correlated with PP, PL, FGP, EGP, SP, GSD, and GYMP. However, GYP in genotype of OW6 exhibited a significantly positive relationship with HD, PH, and GSR. In MC of 2018, there 31 pairs of significantly positive *PCC* values were calculated in six genotypes of OW rice ranged from three pairs in OW4 to nine pairs in OW6. GYP in OW4 was positively correlated with PP, FGP, and GSD. However, the GYP in genotype of OW6 was positively correlated with HD, PH, PP, PL, FGP, EGP, GSR, GW, and GYMP. In RC of 2019, 31 pairs of significantly positive *PCC* values were also calculated in all tested rice ranged from two pairs in OW3 to seven pairs in both OW4 and OW6. The GYP in OW3 was only positively correlated with PP and SP, the GYP in OW4 was positively correlated with PP, PL, EGP, GSR, GSD, GW, and GYMP, the GYP in OW6 was positively correlated with HD, PH, PL, SP, GSD, GW, and GYMP. Within individual genotype across the four years, the number, direction, and size of the *PCC* values between GYP and its components exhibited a series of irregular variations. For example, in genotype of OW3 rice, the number of *PCC* values between GYP and its components was only one in RC of 2016, but four pairs of *PCC* values in MC of both 2017 and 2018, and two pairs of *PCC* values in RC of 2019, respectively. In summary, the number, direction, and size of the *PCC* values between GYP and its eleven components in all tested rice displayed a series of irregular variations and expressed rather stable across the four years.

**Table 3:** *PCCs* between GYP and its components in six genotypes of OW rice.

Genotypes	Crops	HD	PH	PP	PL	FGP	EGP	SP	GSR	GSD	GW	GYMP
		d	cm	n	cm	N			%		g	
OW1	RC2016	0.63**	0.80**	0.96**	0.23**	0.71**	–0.88**	0.45**	0.90**	0.68**	–0.15*	0.54**
OW1	MC2017	–0.41**	–0.48**	0.70**	0.54**	0.56**	0.26**	0.99**	–0.07	0.94**	–0.64**	0.41**
OW1	MC2018	0.44**	0.44**	0.66**	–0.05	0.41**	0.16*	–0.47**	–0.06	–0.08	–0.21**	–0.02
OW1	RC2019	0.72**	0.72**	–0.20**	–0.34**	0.24**	–0.31**	–0.34**	–0.44**	–0.08	–0.47**	0.72**
OW2	RC2016	0.00	–0.53**	–0.48**	–0.26**	–0.10	0.54**	–0.01	–0.58**	0.21**	0.09	–0.17*
OW2	MC2017	0.80**	0.59**	0.74**	–0.29**	–0.12	–0.66**	–0.68**	0.67**	–0.68**	–0.07	–0.32**
OW2	MC2018	0.56**	0.56**	0.34**	–0.17*	–0.51**	–0.19*	0.22**	–0.20**	0.05	0.39**	0.71**
OW2	RC2019	0.58**	0.58**	0.47**	–0.82**	–0.69**	–0.87**	0.17*	–0.82**	0.06	0.92**	0.75**
OW3	RC2016	0.00	–0.41**	0.35**	–0.57**	–0.56**	0.14	–0.44**	–0.62**	–0.19*	–0.12	–0.57**
OW3	MC2017	–0.63**	0.45**	0.91**	–0.41**	–0.71**	0.18*	–0.54**	–0.30**	–0.47**	0.37**	–0.61**
OW3	MC2018	–0.39**	–0.39**	0.39**	0.28**	–0.48**	0.16*	0.58**	0.08	–0.61**	–0.06	–0.26**
OW3	RC2019	–0.93**	–0.93**	0.22**	–0.99**	–0.84**	–0.97**	0.14**	–0.59**	–0.73**	0.00	–0.99**
OW4	RC2016	0.74**	0.34**	–0.62**	0.96**	0.99**	–0.40**	0.82**	–0.97**	0.66**	–0.85**	0.65**
OW4	MC2017	–0.82**	0.41**	0.54**	0.14	–0.30**	0.23**	–0.23**	–0.30**	–0.30**	0.40**	–0.30**
OW4	MC2018	–0.75**	–0.75**	0.64**	–0.38**	0.70**	–0.31**	–0.52**	–0.51**	0.43**	–0.42**	–0.58**
OW4	RC2019	–0.46**	–0.46**	0.15*	0.43**	0.05	0.31**	0.11	0.21**	0.58**	0.69**	0.30**
OW5	RC2016	–0.31**	0.89**	–0.25**	–0.25**	0.99**	–0.29**	0.95**	0.93**	–0.17*	0.77**	0.96**
OW5	MC2017	–0.94**	–0.23**	0.22**	0.26**	–0.96**	0.96**	–0.90**	–0.97**	–0.85**	–0.27**	–0.76**
OW5	MC2018	0.14	0.14	0.22**	0.26**	0.12	0.68**	–0.11	0.07	–0.29**	0.07	0.25**
OW5	RC2019	0.18*	0.16*	0.47**	–0.95**	0.98**	–0.88**	–0.98**	–0.86**	0.03	–0.97**	0.31**
OW6	RC2016	–0.82**	–0.47**	0.10	0.05	0.25**	0.13	0.35**	–0.07	0.44**	0.93**	0.04
OW6	MC2017	0.79**	0.24**	–0.31**	–0.20**	–0.50**	–0.43**	–0.46**	0.39**	–0.47**	–0.07	–0.30**
OW6	MC2018	0.52**	0.67**	0.63**	0.49**	0.59**	0.51**	0.01	0.34**	–0.15*	0.29**	0.79**
OW6	RC2019	0.93**	0.99**	–0.51**	0.95**	–1.00**	–0.85**	0.93**	–0.32**	0.68**	0.85**	0.99**

*and** Significant at the 0.05 and 0.01 probability level, respectively.

*PCCs* are for grain yield of OW rice (*a*_*0.05*_* r* = 0.146; *a*_*0.01*_* r* = 0.192), *PCCs* values without asterisks are nonsignificant.

### Grain shape traits in OW rice

**Figure 3:**
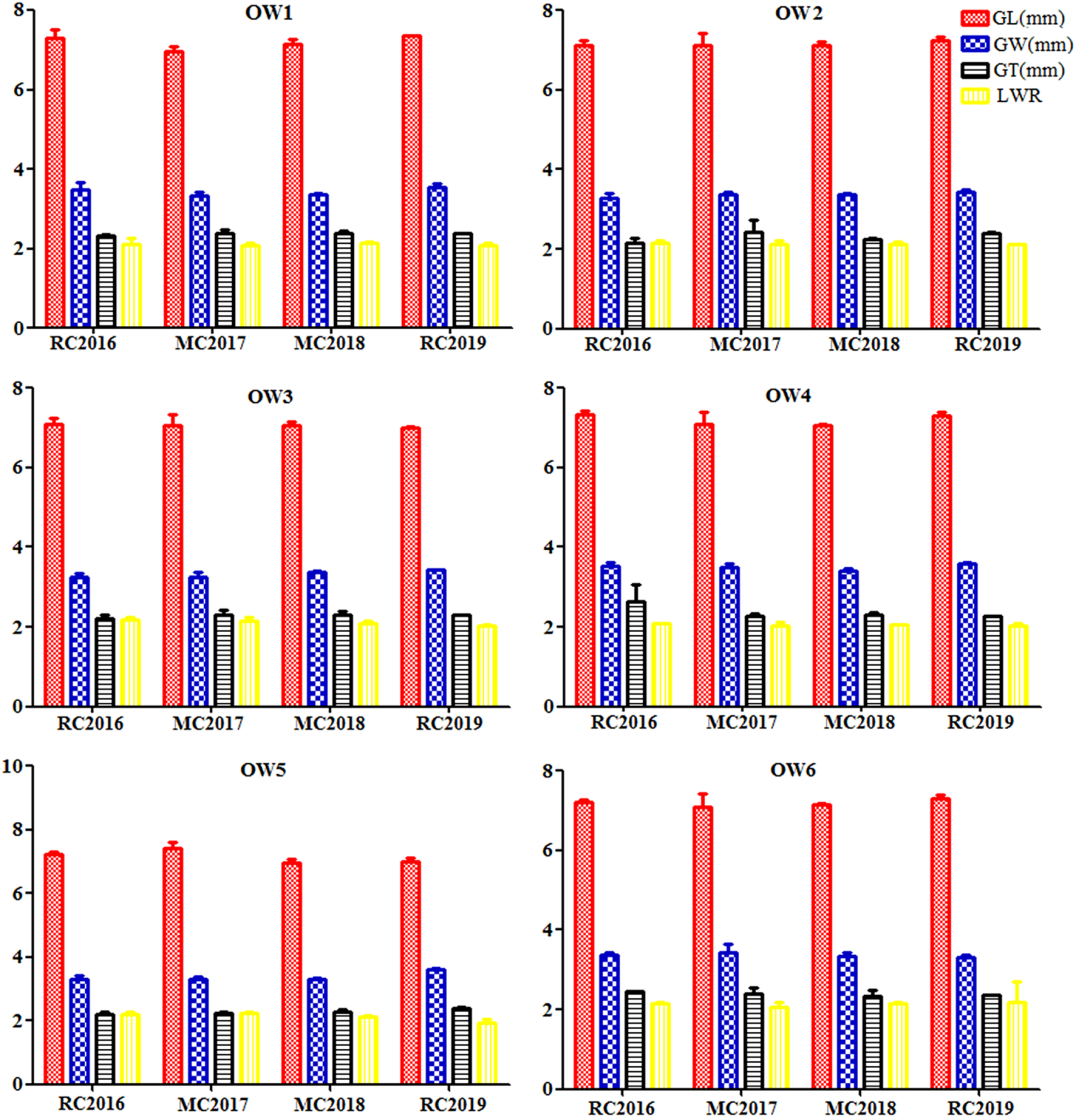
Grain shape traits in six genotypes of OW rice.

Four grain shape traits in six genotypes of OW rice displayed small significant difference and exhibited relatively stable across RC in 2016, MC in 2017, MC in 2018, and RC in 2019 (Table 4). Within individual genotype of OW rice, the four grain shape traits also exhibited small differences across the 4 years (Fig. 3). The GL in four genotypes of OW1, OW2, OW3, and OW6 displayed nonsignificant difference across the 4 years, but exhibited small significant difference in two genotypes of OW4 and OW5. The GW in genotypes of OW3 and OW6 exhibited small differences across the 4 years, but exhibited relatively stable in genotypes of OW1, OW2, OW4, and OW5. The GT in OW1, OW4, and OW6 exhibited nonsignificant differences across the 4 years. The LWR in all tested rice except for OW5 exhibited relative stable across the 4 years. Overall, the field performances on four grain shape traits in partial tested OW rice displayed significant difference and expressed relatively stable across the 4 years.

**Table 4:** Grain shape traits in six genotypes of OW rice.

Genotypes	Crops	GL	GW	GT	LWR
		mm			
OW1	RC2016	7.31 ± 0.21^A^	3.50 ± 0.16^A^	2.33 ± 0.04^A^	2.10 ± 0.16^A^
OW1	MC2017	6.96 ± 0.14^A^	3.33 ± 0.11^A^	2.39 ± 0.10^A^	2.09 ± 0.07^A^
OW1	MC2018	7.14 ± 0.13^A^	3.36 ± 0.03^A^	2.38 ± 0.08^A^	2.13 ± 0.05^A^
OW1	RC2019	7.36 ± 0.01^A^	3.56 ± 0.09^A^	2.38 ± 0.02^A^	2.07 ± 0.08^A^
OW2	RC2016	7.14 ± 0.10^A^	3.29 ± 0.11^A^	2.17 ± 0.12^B^	2.17 ± 0.06^A^
OW2	MC2017	7.13 ± 0.31^A^	3.36 ± 0.09^A^	2.44 ± 0.28^A^	2.12 ± 0.10^A^
OW2	MC2018	7.14 ± 0.08^A^	3.36 ± 0.06^A^	2.24 ± 0.03^B^	2.13 ± 0.05^A^
OW2	RC2019	7.26 ± 0.07^A^	3.44 ± 0.05^A^	2.40 ± 0.04^A^	2.11 ± 0.01^A^
OW3	RC2016	7.09 ± 0.17^A^	3.24 ± 0.09^B^	2.23 ± 0.07^A^	2.19 ± 0.06^A^
OW3	MC2017	7.05 ± 0.29^A^	3.25 ± 0.11^B^	2.32 ± 0.12^A^	2.17 ± 0.08^A^
OW3	MC2018	7.06 ± 0.11^A^	3.36 ± 0.04^AB^	2.32 ± 0.07^A^	2.09 ± 0.05^A^
OW3	RC2019	7.00 ± 0.04^A^	3.44 ± 0.00^A^	2.30 ± 0.01^A^	2.04 ± 0.02^A^
OW4	RC2016	7.34 ± 0.09^A^	3.54 ± 0.07^A^	2.64 ± 0.44^A^	2.08 ± 0.02^A^
OW4	MC2017	7.09 ± 0.30^AB^	3.51 ± 0.08^A^	2.28 ± 0.06^B^	2.02 ± 0.11^A^
OW4	MC2018	7.05 ± 0.05^B^	3.42 ± 0.04^A^	2.32 ± 0.06^B^	2.06 ± 0.01^A^
OW4	RC2019	7.30 ± 0.10^AB^	3.60 ± 0.03^A^	2.28 ± 0.01^B^	2.03 ± 0.07^A^
OW5	RC2016	7.23 ± 0.08^B^	3.31 ± 0.12^A^	2.18 ± 0.11^B^	2.19 ± 0.07^AB^
OW5	MC2017	7.42 ± 0.21^A^	3.32 ± 0.07^A^	2.23 ± 0.05^B^	2.23 ± 0.06^A^
OW5	MC2018	6.98 ± 0.08^AB^	3.32 ± 0.02^A^	2.28 ± 0.06^B^	2.14 ± 0.01^AB^
OW5	RC2019	7.00 ± 0.10^AB^	3.60 ± 0.03^A^	2.40 ± 0.03^A^	1.95 ± 0.08^B^
OW6	RC2016	7.22 ± 0.05^A^	3.38 ± 0.07^AB^	2.45 ± 0.00^A^	2.14 ± 0.06^A^
OW6	MC2017	7.10 ± 0.34^A^	3.44 ± 0.20^A^	2.40 ± 0.14^A^	2.07 ± 0.13^A^
OW6	MC2018	7.16 ± 0.04^A^	3.34 ± 0.09^AB^	2.34 ± 0.14^A^	2.14 ± 0.05^A^
OW6	RC2019	7.32 ± 0.08^A^	3.32 ± 0.04^B^	2.36 ± 0.02^A^	2.20 ± 0.50^A^

Values followed by a different letter are significantly different at 5% (capital) probability levels, respectively.

### *PCCs* for grain shape traits in OW rice

A total 130 pairs of significant *PCC* values for the four grain shape traits exhibited their various numbers, directions, and size in six genotypes of OW rice across the 4 years (Table 5). Among them, the number of pairs of significantly positive *PCC* values was 16 in RC of 2016, 19 in MC of 2017, 13 in MC of 2018, and 15 in RC of 2019, respectively. Within genotype of OW1 rice, GL was positively significant relationship with GW in MC of 2017, MC of 2018, RC of 2019, but negatively significant correlation with GW in RC of 2016. However in genotype of OW4 rice, the GL was positively significant relationship with GW in MC of 2017, but negatively significant relationship with GW across the remaining three seasons. The GW was negatively significant correlation with LWR in all tested rice except for OW3 in RC of 2019. The GT was positively significant correlation with LWR in OW3 in both MC 2017 and RC 2019, but negatively significant relationship with LWR in both RC 2016 and MC 2018. However, GT in OW6 was negatively significant correlation with LWR across the 4 years. The numbers, directions, and size of *PCCs* for the four grain quality traits exhibited a series of irregular variations in all tested rice.

**Table 5:** *PCCs* for grain shape traits in six genotypes of OW rice.

Genotype	Crops	Traits	GL	GW	GT
			mm		
OW1	RC2016	GW (mm)	–0.75**		
OW1	MC2017	GW (mm)	0.38**		
OW1	MC2018	GW (mm)	0.24**		
OW1	RC2019	GW (mm)	0.62**		
OW2	RC2016	GW (mm)	0.41**		
OW2	MC2017	GW (mm)	0.16*		
OW2	MC2018	GW (mm)	0.95**		
OW2	RC2019	GW (mm)	0.98**		
OW3	RC2016	GW (mm)	0.43**		
OW3	MC2017	GW (mm)	0.51**		
OW3	MC2018	GW (mm)	0.26**		
OW3	RC2019	GW (mm)	–0.50**		
OW4	RC2016	GW (mm)	–0.32**		
OW4	MC2017	GW (mm)	0.35**		
OW4	MC2018	GW (mm)	–0.36**		
OW4	RC2019	GW (mm)	–0.22**		
OW5	RC2016	GW (mm)	0.23**		
OW5	MC2017	GW (mm)	0.52**		
OW5	MC2018	GW (mm)	–0.32**		
OW5	RC2019	GW (mm)	–0.55**		
OW6	RC2016	GW (mm)	0.39**		
OW6	MC2017	GW (mm)	0.35**		
OW6	MC2018	GW (mm)	–0.82**		
OW6	RC2019	GW (mm)	0.61**		
OW1	RC2016	GT (mm)	0.54**	0.1	
OW1	MC2017	GT (mm)	–0.10	0.55**	
OW1	MC2018	GT (mm)	–0.42**	0.78**	
OW1	RC2019	GT (mm)	–0.19*	0.65**	
OW2	RC2016	GT (mm)	–0.20**	0.73**	
OW2	MC2017	GT (mm)	–0.27**	0.21**	
OW2	MC2018	GT (mm)	0.98**	0.91**	
OW2	RC2019	GT (mm)	0.90**	0.97**	
OW3	RC2016	GT (mm)	–0.56**	–0.06	
OW3	MC2017	GT (mm)	0.60**	0.20**	
OW3	MC2018	GT (mm)	0.12	0.55**	
OW3	RC2019	GT (mm)	0.41**	0.59**	
OW4	RC2016	GT (mm)	0.75**	0.34**	
OW4	MC2017	GT (mm)	0.31**	0.92**	
OW4	MC2018	GT (mm)	–0.74**	–0.34**	
OW4	RC2019	GT (mm)	–0.95**	0.09	
OW5	RC2016	GT (mm)	–0.90**	–0.62**	
OW5	MC2017	GT (mm)	–0.10	–0.30**	
OW5	MC2018	GT (mm)	–0.27**	–0.61**	
OW5	RC2019	GT (mm)	–0.98**	0.71**	
OW6	RC2016	GT (mm)	–0.15*	0.47**	
OW6	MC2017	GT (mm)	–0.65**	0.14	
OW6	MC2018	GT (mm)	0.02	0.39**	
OW6	RC2019	GT (mm)	0.84**	0.94**	
OW1	RC2016	LWR	0.88**	–0.97**	0.12
OW1	MC2017	LWR	0.20**	–0.83**	–0.66**
OW1	MC2018	LWR	0.82**	–0.35**	–0.86**
OW1	RC2019	LWR	–0.18*	–0.88**	–0.93**
OW2	RC2016	LWR	0.02	–0.90**	–0.91**
OW2	MC2017	LWR	0.80**	–0.45**	–0.37**
OW2	MC2018	LWR	–0.66**	–0.87**	–0.60**
OW2	RC2019	LWR	–0.31**	–0.50**	–0.69**
OW3	RC2016	LWR	0.42**	–0.64**	–0.42**
OW3	MC2017	LWR	0.64**	–0.34**	0.47**
OW3	MC2018	LWR	0.69**	–0.52**	–0.31**
OW3	RC2019	LWR	0.81**	0.10	0.87**
OW4	RC2016	LWR	0.91**	–0.68**	0.43**
OW4	MC2017	LWR	0.19*	–0.85**	–0.80**
OW4	MC2018	LWR	0.68**	–0.93**	–0.02
OW4	RC2019	LWR	0.86**	–0.68**	–0.76**
OW5	RC2016	LWR	0.27**	–0.87**	0.16*
OW5	MC2017	LWR	0.72**	–0.21**	0.14
OW5	MC2018	LWR	0.93**	–0.66**	0.05
OW5	RC2019	LWR	0.93**	–0.81**	–0.99**
OW6	RC2016	LWR	0.32**	–0.75**	–0.58**
OW6	MC2017	LWR	0.42**	–0.70**	–0.65**
OW6	MC2018	LWR	0.87**	–0.99**	–0.29**
OW6	RC2019	LWR	0.32**	–0.56**	–0.25**

* and ** Significant at the 0.05 and 0.01 probability level, respectively.

*PCCs* are for the grain shape traits of OW rice (*a*_*0.05*_*r* = 0.146, *a*_*0.01*_*r* = 0.192), *PCCs* values without asterisks are nonsignificant.

### Grain quality traits in OW rice

**Figure 4:**
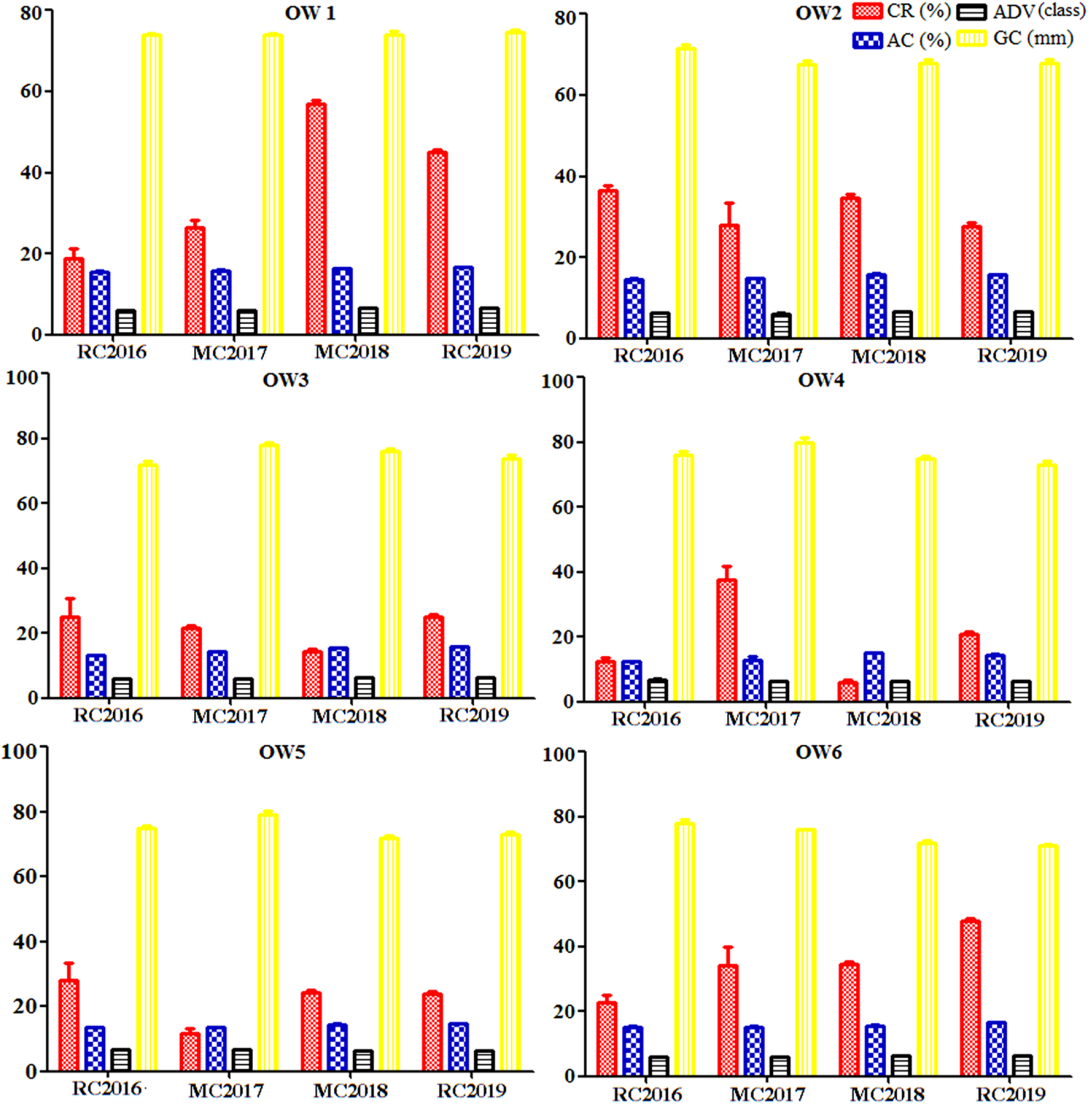
Grain quality traits in six genotypes of OW rice.

Four grain quality traits in six genotypes of OW rice across RC in 2016, MC in 2017, MC in 2018, and RC in 2019 exhibited a relative small significant difference except for the CR (Table 6). In particularly, a series of significant difference on CR was simultaneously observed in all tested rice across the 4 years (Fig. 4; Supplementary Fig. 5). Within individual genotype of OW1 rice, The AC exhibited nonsignificant difference and expressed rather stable across the 4 years, but displayed significant difference on the remaining five genotypes of OW rice. The ADV in genotypes of both OW5 and OW6 displayed nonsignificant difference, but exhibited significant difference in four genotypes of OW1, OW2, OW3, and OW4. The GC displayed nonsignificant difference in three genotypes of OW1, OW4, and OW5, but displayed a relatively small significant difference in three genotypes of OW2, OW3, and OW6. Overall, a series of significant differences on the four grain quality traits were partially observed in all tested rice.

**Table 6:** Grain quality traits in six genotypes of OW rice.

Genotypes	Crops	CR	AC	ADV (class)	GC (mm)
		%			
OW1	RC2016	18.71 ± 2.63^D^	15.50 ± 0.12^A^	6.00 ± 0.01^B^	74.00 ± 0.20^A^
OW1	MC2017	26.28 ± 1.94^C^	15.90 ± 0.12^A^	6.10 ± 0.02^B^	74.00 ± 0.30^A^
OW1	MC2018	57.00 ± 0.71^A^	16.30 ± 0.04^A^	6.50 ± 0.00^A^	74.00 ± 0.83^A^
OW1	RC2019	45.00 ± 0.76^B^	16.60 ± 0.08^A^	6.50 ± 0.00^A^	74.50 ± 0.76^A^
OW2	RC2016	36.49 ± 1.14^A^	14.60 ± 0.19^B^	6.20 ± 0.20^AB^	71.50 ± 0.89^A^
OW2	MC2017	28.10 ± 5.24^B^	14.80 ± 0.17^B^	6.10 ± 0.17^B^	67.60 ± 1.02^AB^
OW2	MC2018	34.75 ± 0.76^A^	15.90 ± 0.08^A^	6.50 ± 0.00^A^	68.00 ± 0.71^B^
OW2	RC2019	27.75 ± 0.76^B^	15.75 ± 0.11^A^	6.50 ± 0.00^A^	68.00 ± 0.71^B^
OW3	RC2016	25.16 ± 5.68^A^	14.20 ± 0.14^BC^	6.00 ± 0.05^B^	72.00 ± 0.89^D^
OW3	MC2017	21.54 ± 0.83^B^	14.30 ± 0.10^BC^	6.00 ± 0.01^B^	78.00 ± 0.75^A^
OW3	MC2018	14.25 ± 0.87^C^	15.45 ± 0.11^AB^	6.50 ± 0.00^A^	76.00 ± 0.83^B^
OW3	RC2019	25.00 ± 0.66^A^	15.9 ± 0.11^A^	6.50 ± 0.00^A^	74.00 ± 0.83^C^
OW4	RC2016	12.44 ± 1.18^C^	12.30 ± 0.14^C^	6.80 ± 0.14^A^	76.00 ± 1.41^A^
OW4	MC2017	37.56 ± 4.42^A^	13.00 ± 0.92^B^	6.20 ± 0.14^B^	80.00 ± 1.45^A^
OW4	MC2018	6.00 ± 0.71^D^	15.00 ± 0.07^A^	6.50 ± 0.00^AB^	75.00 ± 0.71^A^
OW4	RC2019	21.00 ± 0.71^B^	14.50 ± 0.11^B^	6.50 ± 0.00^AB^	73.25 ± 0.87^A^
OW5	RC2016	28.07 ± 5.25^A^	13.50 ± 0.03^AB^	6.70 ± 0.02^A^	75.00 ± 0.89^A^
OW5	MC2017	11.77 ± 1.27^C^	13.50 ± 0.04^B^	6.70 ± 0.01^A^	79.00 ± 1.41^A^
OW5	MC2018	24.25 ± 0.76^B^	14.50 ± 0.11^A^	6.50 ± 0.00^A^	72.00 ± 0.71^A^
OW5	RC2019	23.75 ± 0.76^B^	14.70 ± 0.11^A^	6.50 ± 0.00^A^	73.00 ± 0.71^A^
OW6	RC2016	22.58 ± 2.31^C^	15.30 ± 0.19^B^	6.00 ± 0.01^A^	78.00 ± 1.02^AB^
OW6	MC2017	34.33 ± 5.68^B^	15.30 ± 0.10^B^	6.00 ± 0.02^A^	76.00 ± 0.19^A^
OW6	MC2018	34.75 ± 0.76^B^	15.60 ± 0.11^B^	6.50 ± 0.00^A^	72.00 ± 0.71^B^
OW6	RC2019	47.80 ± 0.77^A^	16.70 ± 0.07^A^	6.50 ± 0.00^A^	71.00 ± 0.63^B^

Values followed by a different letter are significantly different at 5% (capital) probability levels, respectively.

**Figure 5:**
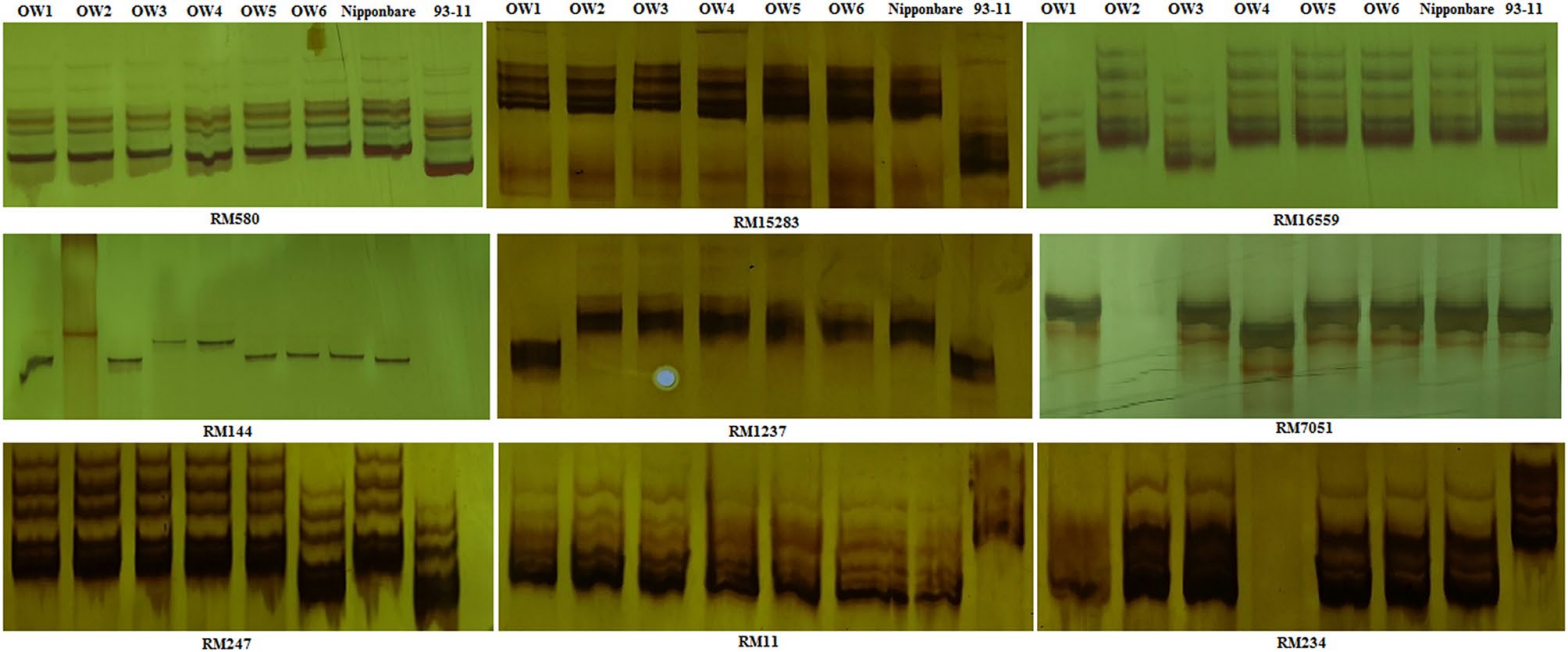
Amplified bands using nine SSR markers with good polyphosim in six OW rice genotypes.

### *PCCs* for grain quality traits in OW rice

Altogether 118 pairs of significant positive *PCC*s for the four grain quality traits exhibiting theirs various numbers, directions, and size in the six genotypes of OW rice across the 4 years (Table 7). Among them, in RC of 2016, there 19 pairs of significantly positive *PCC* values were calculated the six genotypes of OW rice ranged from two pairs of correlations between GC to ADV in OW5 (0.95) and OW6 (0.35) to five pairs for the correlation between CR and AC in all tested rice except for OW5. In MC of 2017, there 13 pairs of significantly positive *PCC* values were calculated and ranged from one pair of PCC values in both OW5 (0.60) and OW6 (0.97) to three pairs for the *PCC* values in OW1 (0.97, 0.30, and 0.35), OW2 (0.45, 0.59, and 0.30), and OW4 (0.32, 0.41, and 0.95) . In MC of 2018, there 23 pairs of significantly positive *PCC* values were calculated and ranged from two pairs of *PCC* values in both OW3 and OW4 to six pairs of *PCC* values in both OW2 and OW6. In RC of 2019, there 20 pairs of significantly positive *PCC* values were calculated and ranged from two pair of relationship between GC and ADV (0.91), AC and CR (0.57) in OW6 to five pairs of correlations among four grain quality traits except for the relationship between AC and ADV in OW5. The numbers, directions, and size of the *PCC* value for grain quality traits displayed a series of irregular variations in all tested rice across the 4 years.

**Table 7:** *PCCs* for grain quality traits among six genotypes of OW rice.

Genotypes	Crops	Traits	CR	AC	ADV (class)
			%		
OW1	RC2016	AC (%)	0.24**		
OW1	MC2017	AC (%)	–0.27**		
OW1	MC2018	AC (%)	0.00		
OW1	RC2019	AC (%)	–0.48**		
OW2	RC2016	AC (%)	0.25**		
OW2	MC2017	AC (%)	0.45**		
OW2	MC2018	AC (%)	0.64**		
OW2	RC2019	AC (%)	0.84**		
OW3	RC2016	AC (%)	0.42**		
OW3	MC2017	AC (%)	–0.82**		
OW3	MC2018	AC (%)	–0.67**		
OW3	RC2019	AC (%)	0.83**		
OW4	RC2016	AC (%)	0.38**		
OW4	MC2017	AC (%)	0.11		
OW4	MC2018	AC (%)	0.00		
OW4	RC2019	AC (%)	–0.53**		
OW5	RC2016	AC (%)	–0.67**		
OW5	MC2017	AC (%)	–0.11		
OW5	MC2018	AC (%)	–0.13		
OW5	RC2019	AC (%)	0.80**		
OW6	RC2016	AC (%)	0.43**		
OW6	MC2017	AC (%)	–0.20**		
OW6	MC2018	AC (%)	0.98**		
OW6	RC2019	AC (%)	0.57**		
OW1	RC2016	ADV(class)	–0.37**	–0.97**	
OW1	MC2017	ADV(class)	–0.26**	0.97**	
OW1	MC2018	ADV(class)	0.63**	0.77**	
OW1	RC2019	ADV(class)	0.53**	–0.63**	
OW2	RC2016	ADV(class)	0.42**	–0.05	
OW2	MC2017	ADV(class)	–0.68**	–0.14	
OW2	MC2018	ADV(class)	0.40**	0.94**	
OW2	RC2019	ADV(class)	0.12	–0.31**	
OW3	RC2016	ADV(class)	0.87**	–0.06	
OW3	MC2017	ADV(class)	–0.03	–0.28**	
OW3	MC2018	ADV(class)	0.13	0.40**	
OW3	RC2019	ADV(class)	–0.48**	–0.32**	
OW4	RC2016	ADV(class)	–0.15*	0.00	
OW4	MC2017	ADV(class)	–0.88**	0.32**	
OW4	MC2018	ADV(class)	–0.95**	0.32**	
OW4	RC2019	ADV(class)	0.51**	0.29**	
OW5	RC2016	ADV(class)	0.85**	–0.31**	
OW5	MC2017	ADV(class)	–0.16*	0.60**	
OW5	MC2018	ADV(class)	0.13	0.80**	
OW5	RC2019	ADV(class)	0.54**	0.05	
OW6	RC2016	ADV(class)	0.34**	0.47**	
OW6	MC2017	ADV(class)	–0.35**	0.12	
OW6	MC2018	ADV(class)	0.98**	1.00**	
OW6	RC2019	ADV(class)	–0.44**	–0.78**	
OW1	RC2016	GC (mm)	0.44**	0.85**	–0.85**
OW1	MC2017	GC (mm)	–0.97**	0.30**	0.35**
OW1	MC2018	GC (mm)	0.85**	0.52**	0.94**
OW1	RC2019	GC (mm)	0.89**	–0.48**	0.83**
OW2	RC2016	GC (mm)	0.23**	0.72**	–0.50**
OW2	MC2017	GC (mm)	–0.13	0.59**	0.30**
OW2	MC2018	GC (mm)	0.85**	0.85**	0.63**
OW2	RC2019	GC (mm)	0.72**	0.38**	0.04
OW3	RC2016	GC (mm)	0.34**	0.95**	–0.09
OW3	MC2017	GC (mm)	–0.59**	0.42**	0.76**
OW3	MC2018	GC (mm)	1.00**	–0.67**	0.13
OW3	RC2019	GC (mm)	–0.08	0.40**	0.63**
OW4	RC2016	GC (mm)	–0.71**	–0.10	–0.30**
OW4	MC2017	GC (mm)	0.41**	0.95**	0.00
OW4	MC2018	GC (mm)	–0.50**	–0.50**	0.32**
OW4	RC2019	GC (mm)	1.00**	–0.53**	0.51**
OW5	RC2016	GC (mm)	0.75**	–0.07	0.95**
OW5	MC2017	GC (mm)	–0.82**	0.00	–0.30**
OW5	MC2018	GC (mm)	–0.85**	0.63**	0.32**
OW5	RC2019	GC (mm)	0.95**	0.95**	0.31**
OW6	RC2016	GC (mm)	–0.49**	–0.56**	0.35**
OW6	MC2017	GC (mm)	–0.21**	0.97**	–0.06
OW6	MC2018	GC (mm)	0.24**	0.32**	0.32**
OW6	RC2019	GC (mm)	–0.10	–0.76**	0.91**

* and ** Significant at the 0.05 and 0.01 probability level, respectively.

*PCCs* for grain quality traits of OW rice (*a*_*0.05*_*r* = 0.146, *a*_*0.01*_*r* = 0.192); the *PCCs* values without asterisks are nonsignificant.

### Genetic diversity of OW rice

**Figure 6:**
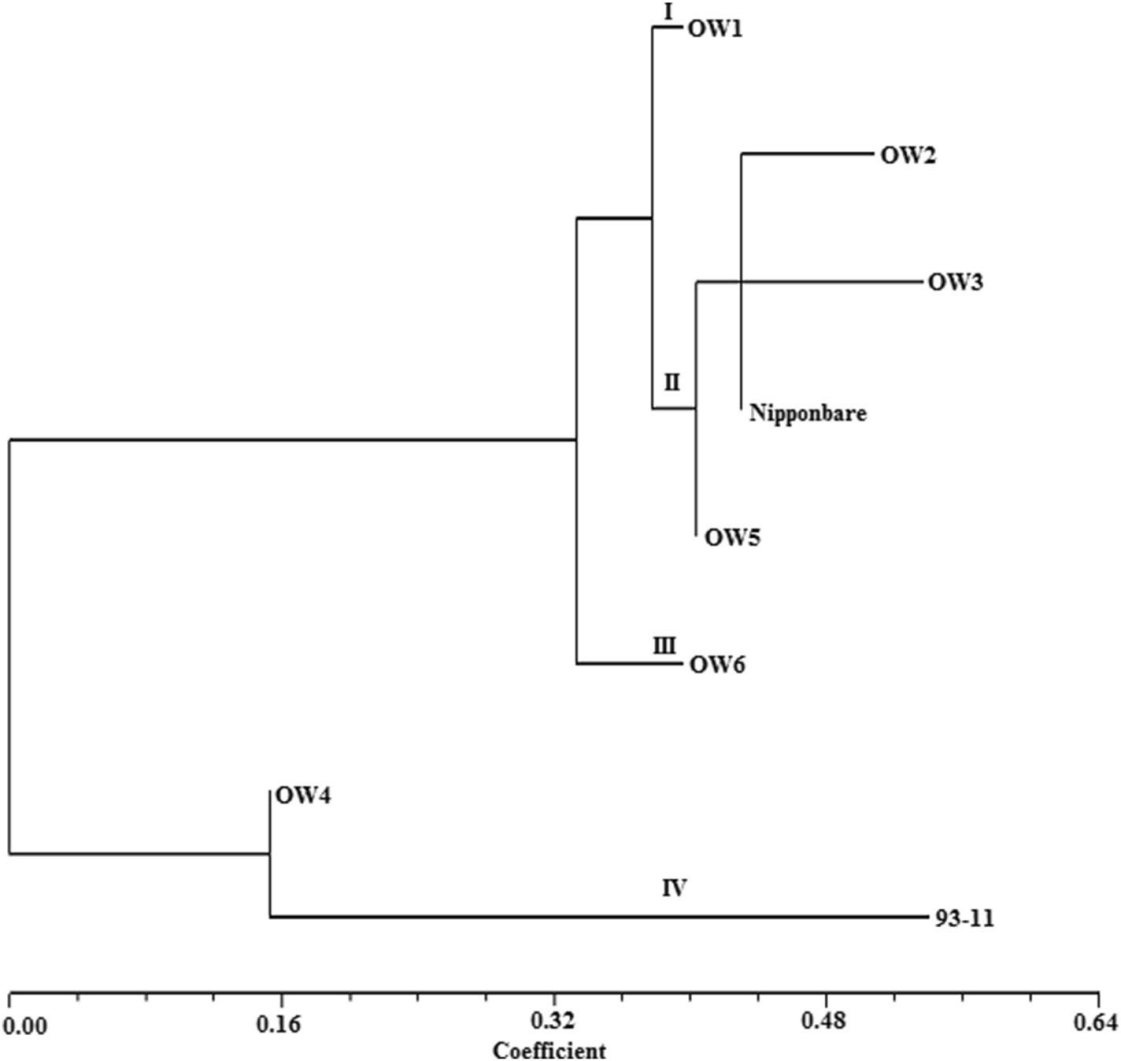
Dendrogram of six genotypes of OW rice based on nine SSR markers.

Six genotypes of OW rice, Nipponbare, and 93-11 were roughly split into four groups at the genetic distance of 0.38 by employing a total of 196 pairs of SSR markers. However, only nine pairs of SSR markers displayed abundant polymorphism, which apparently revealed the molecular differences between six genotypes of OW rice and two sequencing rice varieties (Fig. 5 and 6, Table 8). Genotype of OW1 was separately split into the group I at the genetic distance of 0.384. Genotypes of OW2, OW3, OW5, and Nipponbare were divided into the group II at the genetic distance of 0.40, the group II was split into two subgroups, Genotypes of OW2, OW3, and Nipponbare were split into the 1st subgroup II at the genetic distance of 0.42, OW5 was separately divided into the 2nd subgroup II, Genotypes of OW2, OW3, and Nipponbare could be apparently distinguished at the genetic distance of 0.43. Genotype of OW6 was separately split into the group III at the genetic distance of 0.40. Genotypes of OW4 and 93-11 were divided into the group IV; it was clear to distinguish between 93-11 and OW4 at the genetic distance 0.15. Overall, six genotypes of OW rice, Nipponbare and 93-11 could be apparently distinguished by nine pairs of SSR markers and exhibited abundant genetic diversity at DNA level.

**Table 8:** The SSR primers used in this study.

Chr	SSR	Gp (bp)	Forward primer	Reverse primer	Repeat Motif	Melting temperature (°C)
1	RM580	9,605,625	GATGAACTCGAATTTGCATCC	CACTCCCATGTTTGGCTCC	(CTT)19	55.66/58.43
3	RM15283	1,871,497	GCTACAAATAGCTGCAAACTGC	TTGGACTAGCCTTTGACTGAGG	(AT)14	58.83/59.70
4	RM16559	9,342,920	CCTGGAACCTGGAGGTGTTCTCG	GTCGTGGACGATTTCTTCGTCAGC	(CCG)7	64.60/64.17
4	RM7051	24,116,775	CTCGATGAGCTTGGCGTC	TTCAGTGTTCATCGCCTCTG	(AATC)7	58.30/57.92
5	RM1237	17,956,065	CTCCGCGAGCTTTAGAAGAG	CACATACTCTGGCTCTCCCG	(AG)15	58.17/59.61
7	RM11	19,257,022	TCTCCTCTTCCCCCGATC	ATAGCGGGCGAGGCTTAG	(TC)16	56.97/58.95
7	RM234	25,471,929	ACAGTATCCAAGGCCCTGG	CACGTGAGACAAAGACGGAG	(CT)25	58.69/58.59
11	RM144	28,281,693	TGCCCTGGCGCAAATTTGATCC	GCTAGAGGAGATCAGATGGTAGTGCATG	(ATT)11	64.56/64.34
12	RM247	3,185,581	TAGTGCCGATCGATGTAACG	CATATGGTTTTGACAAAGCG	(CT)16	57.61/53.55

Chromosome abbreviated by Chr.; Genomic position (bp) abbreviated by GP and referred to the whole genome sequence of Nippobare.

## Discussion

### Overwintering cultivated rice resource screened from the existing cultivated rice variety

Developing new genotype of OW rice that combined high yield and good quality across the four seasons will help increase farms’ income by through labor costs saved. However, to screen OW cultivated rice resource was the premise to perform the OW cultivated rice variety breeding project. The present six genotypes of OW cultivated rice resource could survive through natural snowy winter seasons and germinated in the following spring and be harvested after paddy cultivation in the following autumn, which might contain a series of genes or QTLs underlying the OW characteristics, and was prior to be considered as excellent OW rice resources involved into understanding the molecular mechanisms about the OW characteristic ^12^. In particularly, the present six genotypes of OW cultivated rice displayed abundant genetic diversity at the DNA level and were apparently distinguished by employing a total of 196 pairs of SSR markers. However, only nine pairs of SSR markers displayed abundant polymorphism, which apparently revealed the molecular differences between six genotypes of OW rice. More interesting, it may be feasible to integrate OW genes into the current backbone parent of super hybrid rice to develop a novel genotype of OW cultivated rice variety for the future agricultural production and ecological restoration ^24,25^. Therefore, special attention should be given to identification of OW cultivated rice resource in the future rice genetics and breeding project.

### Susceptible GYP and its components in OW rice

For a long time, rice breeders and cultivators have devoted themselves to developing new genotypes of rice with relatively stable field performance on grain yield and quality under different rice cropping systems^26,27^. However, in different genotypes of rice exhibit significant differences in their field performance regarding grain yield and quality traits under different cultivation ecosystem^28,29^. Understanding the field performance variation of GYP and its components in OW rice across the four seasons was the foundation of performing the future OW cultivated rice breeding projects and even commercially released to the farmers^15,16^. Therefore, the present study evaluated the field performance variations on GYP and its components in different genotype of OW rice for the precise identification of stable agronomical traits about OW rice across the 4 years. We observed that the major determinants of GYP, including HD, PP, SP, and GW contributed unequally to GYP in all tested rice across the 4 years. PP in MC of 2018 exhibited a significantly positive correlation with GYP in OW6 but two significantly negative correlations with GYP in OW6 of MC 2017 and RC 2019, respectively. The positive main determinants of GYP in the same OW rice may become negatively contributing components of GYP across the 4 years. These findings were not in agreement with the earlier reports by Huang et al^30^ and Laenoi et al^31^. This phenomenon resulted from a series of significant field performance variations occurred in GYP and its components in six genotypes of OW rice. The field performance on both GYP and its components in all tested rice expressed rather unstable and displayed significant difference across the 4 years and were easily affected by both external environmental factors.

Three important reasons could be applied to explain the reason for that. Firstly, a complicated regulatory network might exist in the grain yield and its components of OW cultivated rice and was strongly influenced by both genotype and environmental factors. Secondly, all genotypes of OW rice identified through natural snowy winter conditions exhibited significant difference on GYP and its components and might possess a set of unique patterns of grain yield and quality related to traits. Thirdly, the grain yield and quality characters in six genotypes of OW rice were controlled by both major gene and polygene and easily affected by multi-external environmental factors of weather conditions, transplanting time, planting density, and fertilization. More interestingly, genotypes OW2 and OW3 exhibited nonsignificant variations in GSR as result of being insensitive to daily Max T (°C) during each ripening period even if the significant difference on daily Max T (°C) occurred at the same period across the 4 years. This finding is in agreement with the previous report by Ishimaru et al^32^. The field performance values for HD, PH, and PP in all tested rice in RC of both 2016 and 2019 were bigger than those in MC of both 2017 and 2018. Consequently, these regular variations in the seasonal response of GYP and its components in all tested OW rice should be given more attention during the future development of OW cultivated rice variety.

### Relatively stable grain shape and grain quality traits in OW rice

The GL, GW, GT, and LWR are important indicators for grain weight and final yield judgment and influence the yield and quality ^33^. Grain shape traits have been widely accepted as complex traits controlled by multiple genes with small genetic effects ^34,35^. In all tested rice across the 4 years, the field performance on GL in OW4 and OW5 and GW in OW2 and OW6 only displayed a relative small significantly different, the GT in OW4 and OW5 and the LWR in OW5 exhibited a small significantly different. The four grain shape traits expressed relative stable in partial OW cultivated rice across the 4 years. This result also suggests that seasonal variation had small impact on grain shape variations. Similar results were reported by Wan et al. ^36^ and Fan et al. ^37^. The grain shape traits of OW rice exhibited a relatively stable field performance through four different seasons and should not be given more attention in the future development of the OW cultivated rice variety.

Four grain quality traits including CR, AC, ADV, and GC have been widely considered as important indicators of grain quality for the new rice variety breeding ^38^. Among them, the CR was the most important determinant of grain appearance quality in rice production ^39,40^. These previous studies have reported that the CR was a complex quantitative trait that was controlled by polygenes and easily influenced by environmental factors ^41,42^. In the present study, the CR in all tested OW rice exhibited significant difference and expressed rather unstable across the 4 years, the CR in all tested rice might be affected by daily Max T, °C during each ripening period for the rice grain filling ^43,44^. The other three quality traits in all tested rice exhibited a relative small significant difference across the 4 years and not were easily affected by both genotype and genotype × environment, which might be regulated by the Waxy gene ^45,46^. The numbers, directions, and size of *PCC* values for the four grain quality traits displayed a series of irregular variations and were strongly affected by both genotype and genotype × environment. Similar results have been reported in previous studies^47,48^. Consequently, special attention should be given to the chalkiness rate in the future OW cultivated rice improvement program. Meanwhile, the remaining three grain quality traits displayed nonsignificant difference on partial tested rice across the 4 years, this was suggested that some gain quality traits in some OW cultivated rice variety could expressed rather stable and were insensitivity to external environmental factors across the 4 years. Consequently, we should be given more attention to the stable grain quality traits in the future development of the OW cultivated rice variety.

## Supplementary Information

Supplementary Figure 1.

Supplementary Figure 2.

Supplementary Figure 3.

Supplementary Figure 4.

Supplementary Figure 5.

Supplementary Legends.
