# Supplementary figures and images for: Field performance on grain yield and quality and genetic diversity of overwintering cultivated rice (Oryza sativa L.) in southwest China
Source: Sci Rep. 2021 Jan 19;11:1846. doi: 10.1038/s41598-021-81291-8 (PMC7815827; doi:10.1038/s41598-021-81291-8)

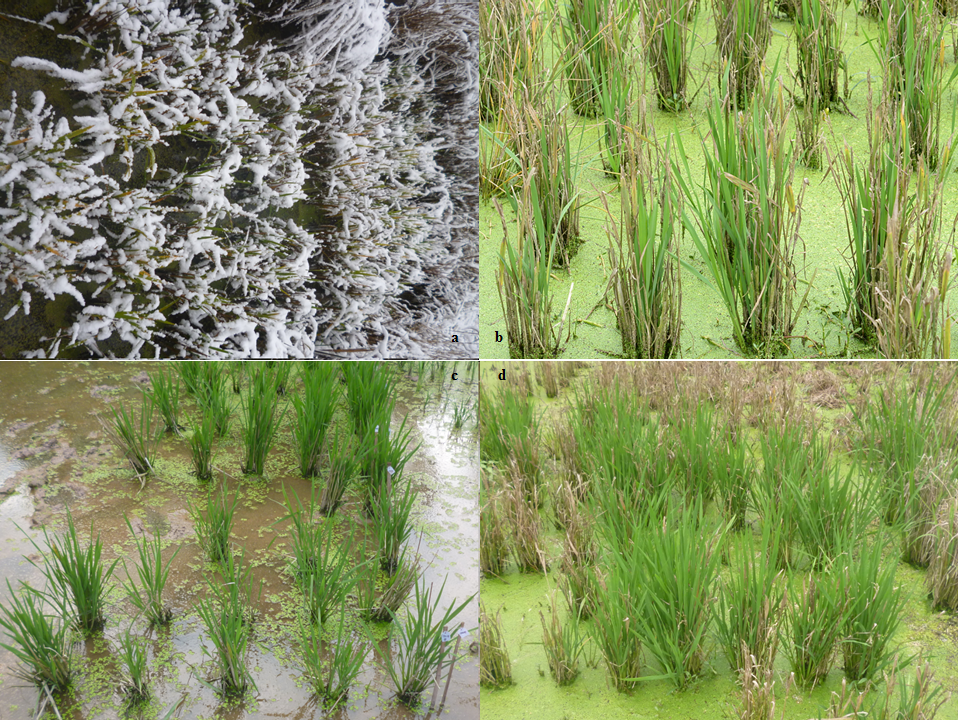

Supplement: Supplementary file 1 — Supplementary Figure 1. [file 41598_2021_81291_MOESM1_ESM.tif]

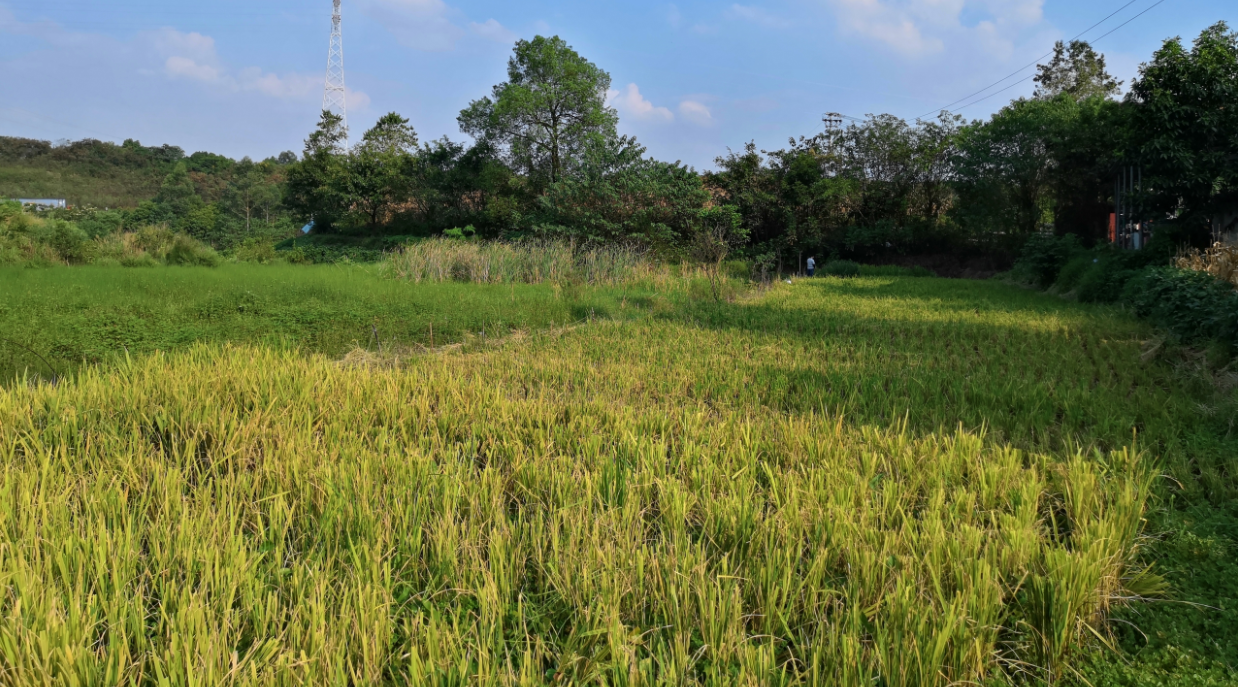

Supplement: Supplementary file 2 — Supplementary Figure 2. [file 41598_2021_81291_MOESM2_ESM.tif]

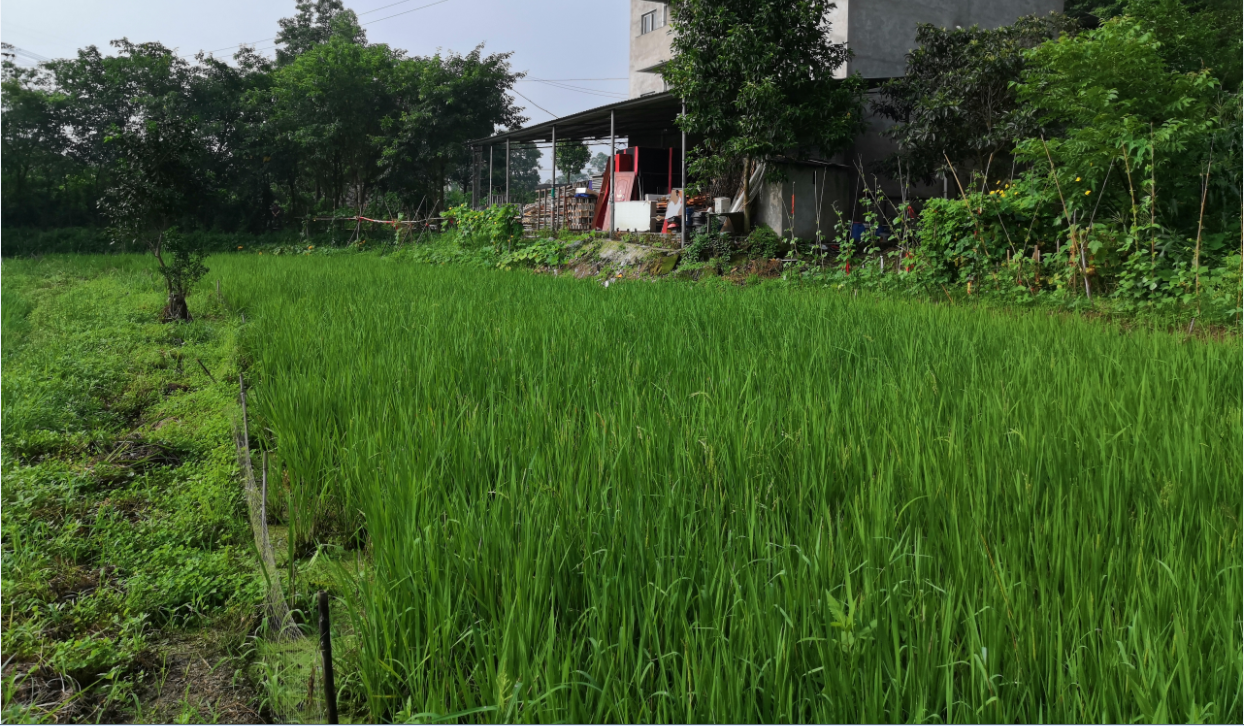

Supplement: Supplementary file 3 — Supplementary Figure 3. [file 41598_2021_81291_MOESM3_ESM.tif]

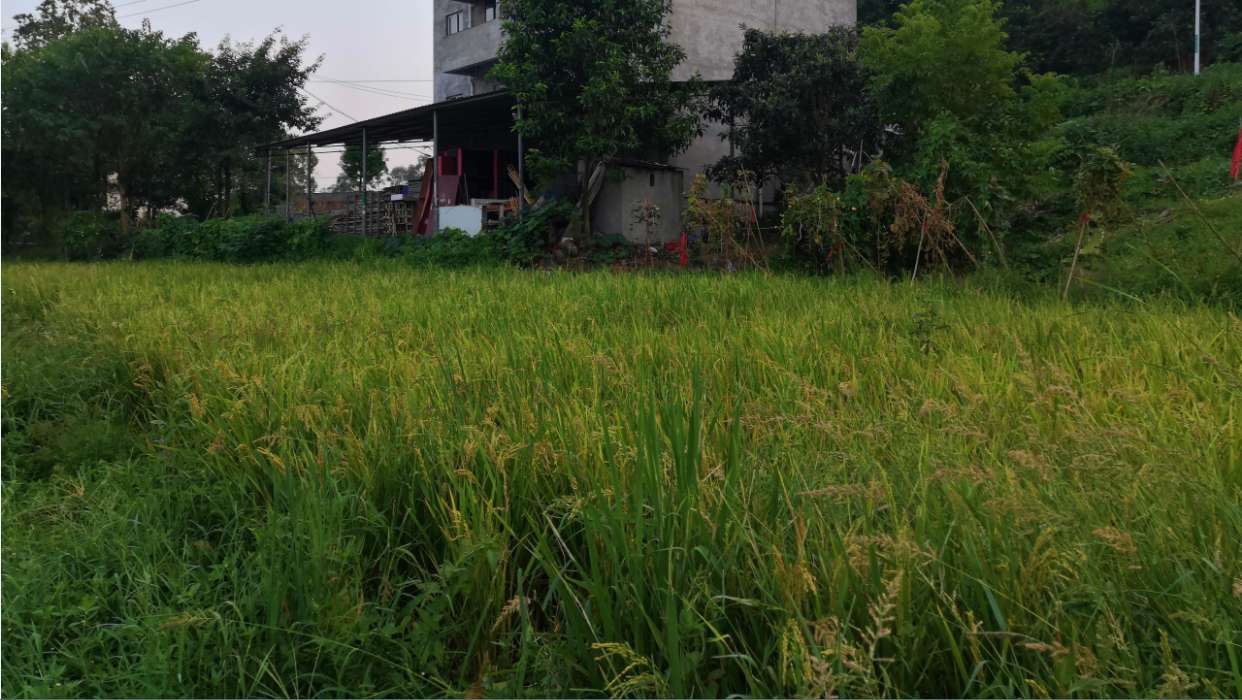

Supplement: Supplementary file 4 — Supplementary Figure 4. [file 41598_2021_81291_MOESM4_ESM.tif]

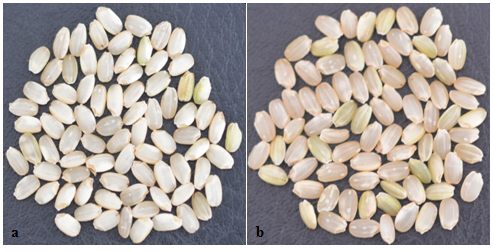

Supplement: Supplementary file 5 — Supplementary Figure 5. [file 41598_2021_81291_MOESM5_ESM.tif]
