# Supplementary material for: Field performance on grain yield and quality and genetic diversity of overwintering cultivated rice (Oryza sativa L.) in southwest China
Source: Sci Rep. 2021 Jan 19;11:1846. doi: 10.1038/s41598-021-81291-8 (PMC7815827; doi:10.1038/s41598-021-81291-8)
Supplement: Supplementary file 6 — Supplementary Legends. [file 41598_2021_81291_MOESM6_ESM.docx]

Field performance on grain yield and quality and genetic diversity of overwintering cultivated rice (*Oryza sativa* L.) in southwest China

Yongshu Liang^*^ (1^st^)

^*^Correspondence should be addressed to Yongshu Liang Email:Yongshuliang@yeah.net

Institution: Chongqing Normal University, China

Address: Chongqing Normal University, University Town, Shapingba District, Chongqing, P. R. China, Post code: 401331

Wen-in Nan (2^nd^)

Email: nanwenbin513@163.com

Institution: Chongqing Normal University, China

Address: Chongqing Normal University, University Town, Shapingba District, Chongqing, P. R. China, Post code: 401331

Xiaojian Qin (3^rd^)

Email qinxiaojian_ab@126.com

Institution: Chongqing Normal University, China

Address: Chongqing Normal University, University Town, Shapingba District, Chongqing, P. R. China, Post code: 401331

Hanma Zhang (4^th^)

Email:15909345392@126.com

Institution: Chongqing Normal University, China

Address: Chongqing Normal University, University Town, Shapingba District, Chongqing, P. R. China, Post code: 401331

Supplementary information:

Supplementary Fig S1 a-d. Field performance of overwintering cultivated rice from snow winter of 2016 to the summer of 2017 in Chongqing, southwest China.

Note: a: OW rice under-snowing in January 2016; b: OW germinated in April 2016; c: OW rice transplanted in May 2016; d: OW rice tillered in May 2016.

Supplementary Fig S2. Field performance on the inaba of overwintering cultivated rice after being harvested in August 2018 in Chongqing, southwest China.

Supplementary Fig S3. Field performance on the tillering of overwintering cultivated rice in June 2019 in Chongqing, southwest China.

Supplementary Fig S4. Field performance on the mature overwintering cultivated rice in September 2019 in Chongqing, southwest China.

Supplementary Fig S5 a-b. Chalkiness rate of OW 4 in RC (a) of 2016 and MC (b) of 2017, Chongqing, Southwest China.
